# Treatment patterns and outcomes in older women with early breast cancer: a population-based cohort study in China

**DOI:** 10.1186/s12885-021-07947-w

**Published:** 2021-03-05

**Authors:** Xu Liu, Dan Zheng, Yanqi Wu, Chuanxu Luo, Yu Fan, Xiaorong Zhong, Hong Zheng

**Affiliations:** 1 Laboratory of Molecular Diagnosis of Cancer, Clinical Research Center for Breast, West China Hospital, Sichuan University, Chengdu, China; 2 Department of Head, Neck and Mammary Gland Oncology, Cancer Center, West China Hospital, Sichuan University, Chengdu, China

**Keywords:** Breast cancer, Elderly women, Adjuvant therapy, Overall survival

## Abstract

**Background:**

Despite the proportion of elderly breast cancer patients has been consistently increasing, the optimal treatment modalities for this population have not been well explored. We summarized the treatment outcomes of these patients in our hospital.

**Methods:**

Older patients with early breast cancer were identified from the Breast Cancer Information Management System at West China Hospital, Sichuan University (2000–2019). We compared tumor characteristics and treatment outcomes between the older group (65–74 years old) and the elderly group (≥75 years old). The Kaplan-Meier and Cox regression analysis were conducted to determine significant prognostic factors.

**Results:**

In total, 1094 patients were included. The median follow-up time for this cohort was 59 months. The majority of patients underwent surgery and benefited from surgical treatment. Elderly group patients were less likely to receive adjuvant chemotherapy or postmastectomy radiotherapy (PMRT) compared to the older group. However, adjuvant chemotherapy was associated with improved overall survival (OS) (hazard ratio [HR] 0.521, 95% confidence interval [CI] 0.284–0.955, *P* = 0.035). Subgroup analysis revealed that patients with grade III disease best benefited from adjuvant chemotherapy. PMRT offered a significant improvement in local disease control, but not in OS. Furthermore, endocrine therapy improved the OS of HR-positive patients (HR 0.440, 95%CI 0.261–0.741, *P* = 0.002), especially for cases aged 65–74 years. Also, receipt of trastuzumab in HER2-positive patients was associated with better OS (HR 0.168, 95%CI 0.029–0.958, *P* = 0.045).

**Conclusions:**

Our findings suggest that surgery, adjuvant chemotherapy, endocrine and targeted therapy are associated with improved OS in older breast cancer patients. Moreover, clinicopathological characteristics should be comprehensively considered when making treatment decisions for these patients.

**Supplementary Information:**

The online version contains supplementary material available at 10.1186/s12885-021-07947-w.

## Background

Breast cancer is the most common malignancy in women worldwide, and the incidence rates have been rising for most countries in transition over the last decades [1]. From 2011 to 2015, the number of new breast cancer cases occurred in Chinese female increased from 248,620 to 304,000, and the age-specific incidence rates peaked at the age group of 55–60 [2, 3]. During the last three decades, the peak of incidence has been gradually shifting to older age group, 27.0% of patients with breast cancer are estimated to be 65 years or older in China by 2030 [4, 5].

However, despite the increasing proportion of elderly patients with breast cancer, data on treatments and outcomes for this patient population are limited, partly because elderly patients have been under-represented in most breast cancer treatment trials [6]. Previous studies have even yielded inconsistent results, leading to challenges for clinicians in managing elderly patients. For instance, some studies showed that reduced treatment did not impact survival outcomes [7], while others found that decreased survival was associated with the under-treatment in elderly breast cancer patients [8, 9]. Moreover, the guidelines for their younger counterparts are difficult to extrapolate to older patients, who need to be considered with multiple factors in decision-making, such as functional status, comorbidities, and life expectancy. More researches on breast cancer in older people are needed to improve outcomes and prepare for extensive demand for treatment of this rapidly growing population [10].

Therefore, this study aimed to investigate the tumor characteristics and the associations between treatment modalities and survival in a large population-based cohort of patients over 65 years of age with early breast cancer.

## Methods

### Study population

Breast cancer information management system (BCIMS) of West China Hospital, Sichuan University, contains over 16,000 breast cancer patient cases dating back to 1989 and prospectively records demographic and clinicopathologic characteristics, medical history, diagnosis, laboratory results and treatments, as described previously [11]. Female breast cancer patients diagnosed between 2000 and 2019 and who were over 65 years of age at initial diagnosis were identified from BCIMS (*n* = 1145). Patients with synchronous distant metastases (*n* = 44) or those lacking essential registry data (*n* = 7) were excluded. In total, 1094 consecutive female patients were included in this cohort. Ethical approval for this study was provided by the Clinical Test and Biomedical Ethics Committee (approval number:2012130), West China Hospital, Sichuan University. And all methods were performed in accordance with the Declaration of Helsinki. Informed consent was obtained from all patients at the time of initial diagnosis.

### Data collection

Demographic, clinicopathologic, and treatment information were derived from the BCIMS. Demographic records included age at diagnosis and body mass index (BMI). The BMI cutoffs are based on the standards recommended in the Guidelines for Prevention and Control of Overweight and Obesity in Chinese Adults [12]. Clinicopathological factors included tumor histology, T stage, axillary lymph node status, clinical stage, hormone receptor (HR) status, Human epidermal growth factor receptor-2 (HER2) status, Ki-67 index and tumor grade. The positivity of estrogen receptor (ER) and progesterone receptor (PR) was determined by immunohistochemistry (IHC) according to the Guidelines for Testing of ER and PR in Breast Cancer [13]. HR was considered positive if either ER or PR was positive. HER2 status was determined by IHC and fluorescence in situ hybridization (FISH) following the Guidelines for HER2 Detection in Breast Cancer [14]. The threshold of Ki-67 was positivity in 20% of cells: low Ki-67 [≤ 20%], high Ki-67 [> 20%]. Histological tumor grade was evaluated by the Nottingham grading system and divided into the following categories: grade I, grade II and grade III. The treatment modalities included surgery, neoadjuvant chemotherapy, adjuvant chemotherapy, radiotherapy, endocrine therapy, and targeted therapy.

### Follow-up and study outcomes

The follow-up was conducted every 4 months in the first 3 years after diagnosis, every 6 to 12 months in the fourth and fifth years, and annually after 5 years. Follow-up was operated by office visit, telephone, email, or postal contact, as described in our previous report [15]. Lost to follow-up was defined as failure to contact with the patient on > 2 consecutive occasions.

Overall survival (OS) was calculated as the time from the date of diagnosis to the date of death from any cause. Disease free survival (DFS) was defined as the time from diagnosis to the first event (death from any cause, locoregional recurrence, distant metastasis and second primary breast cancer). Locoregional recurrence (LRR) was defined as recurrence in the ipsilateral chest wall, supraclavicular and infraclavicular areas, axilla, and internal mammary region. Locoregional recurrence-free survival (LRFS) was counted from surgery date to the date of LRR as the first event [16]. Patients alive without any cancer recurrence were censored on the date of the last follow-up.

### Statistical analysis

Data are presented as medians or proportions. Descriptive statistics were used to analyze the clinical and demographic characteristics of the population. According to the nature of the variable, the Chi-square test, Fisher’s exact test or Student’s t-test was used to compare the distribution of baseline characteristics among age groups. Survival curves were estimated using the Kaplan-Meier method, and log-rank tests were performed for comparison of survival outcomes. Cox proportional hazards regression models were used to determine the significant prognostic factors of survival and expressed as hazard ratio (HR) with 95% confidence interval (CI). Missing data were processed using multiple imputation. All statistical tests were 2-tailed and *P* values less than 0.05 were considered to be statistically significant. All statistical analyses were performed using IBM SPSS statistics software (version 23.0) and R software (version 3.5.1; R Foundation).

## Results

### Patients characteristics

A total of 1094 women with early-stage breast cancer were included in the analysis. The median age at diagnosis of breast cancer was 68 years (range 65–94 years) for the whole patients. The demographic and tumor characteristics of patients are summarized in Table 1. The majority of patients had normal BMI (40.2%). Overall, invasive ductal carcinoma was the most common histological type (78.9%). And most of tumors were diagnosed at stage II (46.7%), with positive HR status (69.2%) and negative HER2 status (70.9%).

**Table 1:** Demographic and tumor characteristics of patients by age group

Characteristics	Total	Older group	Elderly group	*P*
	*N* = 1094	*N* = 930	*N* = 164	
	n (%)	n (%)	n (%)	
Age at diagnosis (years)				
Median (range)	68 (65–94)	68 (65–74)	77 (75–94)	
Body mass index (Kg/m^2^)				< 0.001
Underweight (< 18.5)	48 (4.4)	33 (3.5)	15 (9.1)	
Normal (18.5–23.9)	440 (40.2)	370 (39.8)	70 (42.7)	
Overweight (24.0–27.9)	310 (28.3)	278 (29.9)	32 (19.5)	
Obese (≥ 28.0)	94 (8.6)	86 (9.2)	8 (4.9)	
Unknown^a^	202 (18.5)	163 (17.5)	39 (23.8)	
Histology				0.248
DCIS	86 (7.9)	68 (7.3)	18 (11.0)	
IDC	863 (78.9)	739 (79.5)	124 (75.6)	
Other	97 (8.9)	81 (8.7)	16 (9.8)	
Unknown^a^	48 (4.4)	42 (4.5)	6 (3.7)	
Clinical stage				0.440
0-I	269 (24.6)	223 (24.0)	46 (28.0)	
II	511 (46.7)	441 (47.4)	70 (42.7)	
III	269 (24.6)	230 (24.7)	39 (23.8)	
Unknown^a^ (I-III)	45 (4.1)	36 (3.9)	9 (5.5)	
T stage				0.455
≤ T1	394 (36.0)	333 (35.8)	61 (37.2)	
T2	525 (48.0)	454 (48.8)	71 (43.3)	
T3-T4	125 (11.4)	103 (11.1)	22 (13.4)	
Unknown^a^	50 (4.6)	40 (4.3)	10 (6.1)	
N stage				0.109
N0	583 (53.3)	485 (52.2)	98 (59.8)	
N1	302 (27.6)	260 (28.0)	42 (25.6)	
N2	112 (10.2)	103 (11.1)	9 (5.5)	
N3	97 (8.9)	82 (8.8)	15 (9.1)	
HR status				0.815
Positive	757 (69.2)	641 (68.9)	116 (70.7)	
Negative	305 (27.9)	260 (28.0)	45 (27.4)	
Unknown^a^	32 (2.9)	29 (3.1)	3 (1.8)	
HER2 status				0.127
Positive	153 (14.0)	137 (14.7)	16 (9.8)	
Negative	776 (70.9)	658 (70.8)	118 (71.9)	
Unknown^a^	165 (15.1)	135 (14.5)	30 (18.3)	
Subtype				0.329
HR + HER2-	584 (53.4)	499 (53.7)	85 (51.8)	
HR + HER2+	76 (6.9)	68 (7.3)	8 (4.9)	
HR-HER2+	77 (7.0)	69 (7.4)	8 (4.9)	
HR-HER2-	188 (17.2)	155 (16.7)	33 (20.1)	
Unknown^a^	169 (15.4)	139 (14.9)	30 (18.3)	
Ki-67 level				0.654
Low (≤ 20%)	466 (42.6)	400 (43.0)	66 (40.2)	
High (> 20%)	467 (42.7)	396 (42.6)	71 (43.3)	
Unknown^a^	161 (14.7)	134 (14.4)	27 (16.5)	
Grade				0.594
I-II	337 (30.8)	292 (31.4)	45 (27.4)	
III	373 (34.1)	318 (34.2)	55 (33.5)	
Unknown^a^	384 (35.1)	320 (34.4)	64 (39.0)	

*Abbreviations*: *DCIS* ductal carcinoma in situ, *IDC* invasive ductal carcinoma, *HR* hormone receptor, *HER2* human epidermal growth factor receptor 2

a: Unknown values were excluded during chi-square tests

All patients were categorized into two subgroups based on their age at diagnosis: the older group (65–74 years old) versus the elderly group (≥ 75 years old). The total number of older group patients was 930 (85.0%), and that of elderly group was 164 (15.0%). The elderly group patients were more likely to be underweight when compared with the older group (*P* <  0.001). No significant differences were found between two age groups in terms of tumor histological type, clinical stage, T stage, N stage, HR status, HER2 status, molecular subtype, Ki-67 level and tumor grade.

### Follow-up and survival outcomes

The median follow-up time for this cohort was 59 months. Death events differed between the two groups, 102 (11.0%) cases died in the older group, and 41 (25.0%) cases died in the elderly group during the follow-up period (*P* <  0.001). However, recurrence and metastasis rate was not different between the two groups (9.1% vs 10.4%, *P* = 0.619).

In this cohort, the 5-year and 10-year OS rates of all patients were 89.0% and 77.5%. Table 2 shows the results of univariate and multivariate analysis of OS in all patients. In the univariate analysis, patients who aged over 75 years, had underweight, diagnosed at stage III, negative for HR status, with high Ki-67 level or with poorly differentiated tumor grade showed trends toward worse survival (all *P* <  0.05). Whereas, in the multivariate analysis, only age older than 75 years (HR = 1.935, 95% CI 1.109–3.376, *P* = 0.020), underweight (HR = 3.262, 95% CI 1.526–6.974, *P* = 0.002), stage III (HR = 6.215, 95% CI 2.713–14.235, *P* <  0.001) and high Ki-67 level (HR = 1.900, 95% CI 1.065–3.388, *P* = 0.030) were independently associated with poor survival. Furthermore, we performed multivariate Cox regression analysis for DFS and found only underweight and stage III were independent risk factors of DFS for the entire population (Additional file 1: Table S1).

**Table 2:** Univariate and multivariate analysis of overall survival in all breast cancer patients

Variable	Univariate analysis		Multivariate analysis	
	HR^a^ (95% CI)	*P*-value	HR^a^ (95% CI)	*P*-value
Age, years				
65–74	1		1	
≥ 75	2.074 (1.442–2.983)	< 0.001	1.935 (1.109–3.376)	0.020
BMI, kg/m^2^				
18.5–23.9	1		1	
< 18.5	2.995 (1.586–5.655)	0.001	3.262 (1.526–6.974)	0.002
> 23.9	1.161 (0.731–1.844)	0.527	0.918 (0.498–1.693)	0.784
Histology				
DCIS	1			
IDC	1.611 (0.817–3.177)	0.169		
Other	1.313 (0.544–3.169)	0.545		
Clinical stage				
0-I	1		1	
II	1.172 (0.704–1.951)	0.542	1.089 (0.453–2.616)	0.850
III	5.275 (3.259–8.538)	< 0.001	6.215 (2.713–14.235)	< 0.001
HR status				
Negative	1		1	
Positive	0.672 (0.471–0.958)	0.028	0.748 (0.442–1.269)	0.282
HER2 status				
Negative	1			
Positive	1.167 (0.685–1.990)	0.570		
Ki-67 level				
Low (≤ 20%)	1		1	
High (> 20%)	2.102 (1.441–3.064)	< 0.001	1.900 (1.065–3.388)	0.030
Grade				
I-II	1		1	
III	1.747 (1.105–2.760)	0.017	1.047 (0.582–1.883)	0.879

### Treatment patterns

#### Surgery

The majority of patients (1043/1094, 95.3%) in this cohort underwent surgery (Table 3). Surgery rates were high for both groups (95.1% vs. 97.0%, respectively, *P* = 0.288). Of those who underwent surgery, 963 patients (92.3%) received modified radical mastectomy, while 54 patients (5.2%) received radical mastectomy, very few patients (*n* = 26, 2.5%) were treated with breast conserving surgery or palliative surgery.

**Table 3:** Treatment patterns in older breast cancer patients

Treatment	Total	Older group	Elderly group	*P*
	N (%)	N (%)	N (%)	
Surgery (n = 1094) ^a^				0.288
Yes	1043 (95.3)	884 (95.1)	159 (97.0)	
No	51 (4.7)	46 (4.9)	5 (3.0)	
Adjuvant chemotherapy (*n* = 1043) ^b^				< 0.001
Yes	717 (68.7)	660 (74.7)	57 (35.8)	
No	326 (31.3)	224 (25.3)	102 (64.2)	
Neoadjuvant chemotherapy (n = 734) ^b^				0.063
Yes	90 (12.3)	83 (13.2)	7 (6.7)	
No	644 (87.7)	547 (86.8)	97 (93.3)	
Radiotherapy (n = 453) ^b^				0.006
Yes	134 (29.6)	126 (31.8)	8 (14.0)	
No	319 (70.4)	270 (68.2)	49 (86.0)	
Endocrine therapy in HR+ patients (*n* = 732) ^b^				0.978
Yes	599 (81.8)	505 (81.8)	94 (81.7)	
No	133 (18.2)	112 (18.2)	21 (18.3)	
Anti-HER2 therapy in HER2+ patients (n = 143) ^b^				0.609
Yes	53 (37.1)	48 (37.8)	5 (31.3)	
No	90 (62.9)	79 (62.2)	11 (68.8)	

*HR* hormone receptor, *HER2* human epidermal growth factor receptor 2

a: All patients were included in the analysis

b: Only operated patients were included in the analysis

**Fig. 1:**
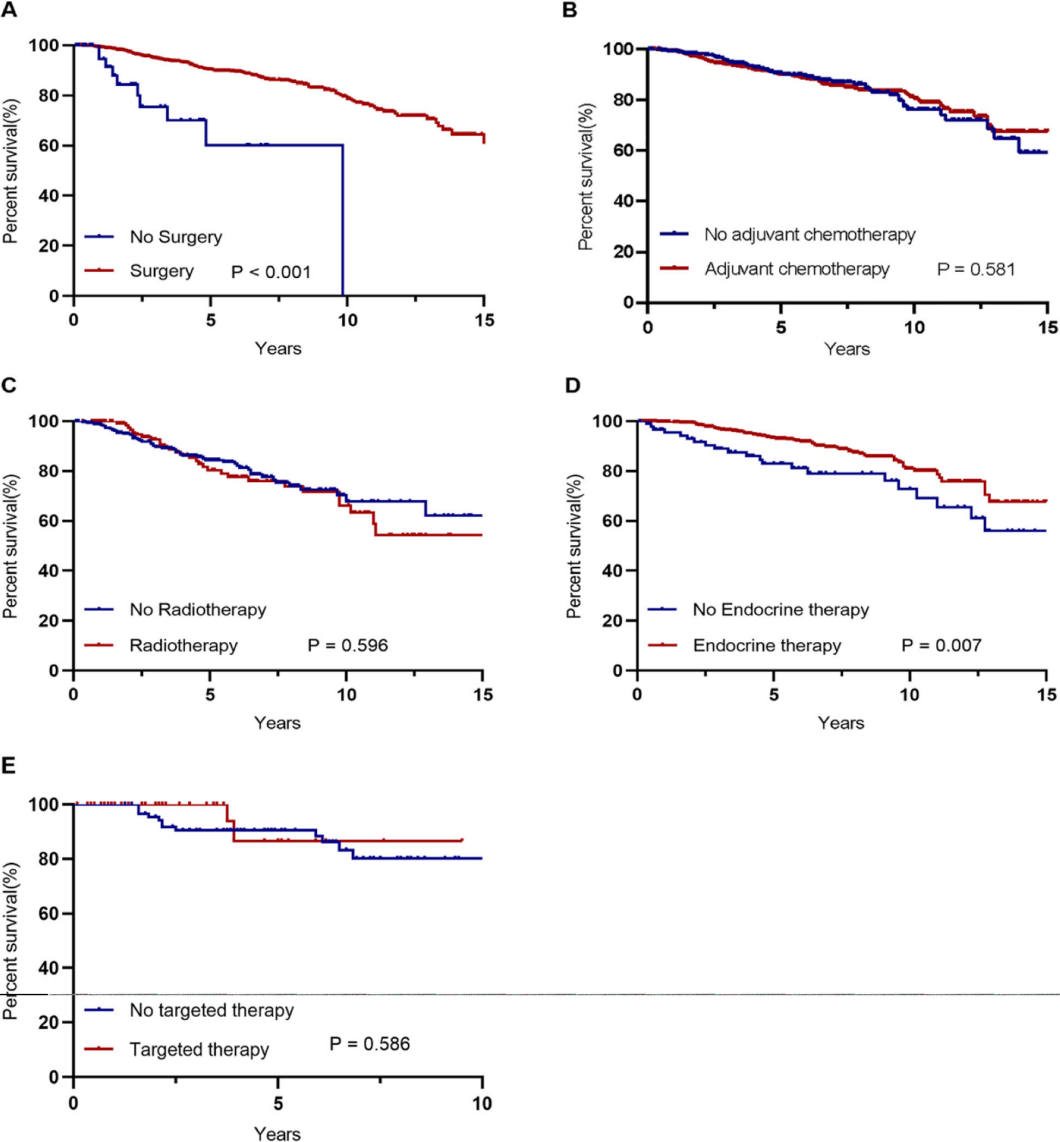
Kaplan–Meier estimates of overall survival by treatment modalities. **a**: surgical treatment in all patients; **b**: chemotherapy in post-operative patients; **c**: postmastectomy radiotherapy in patients with lymph node-positive tumors; **d**: endocrine therapy in patients with HR-positive tumors; **e**: targeted therapy in patients with HER2-positive tumors.

**Fig. 2:**
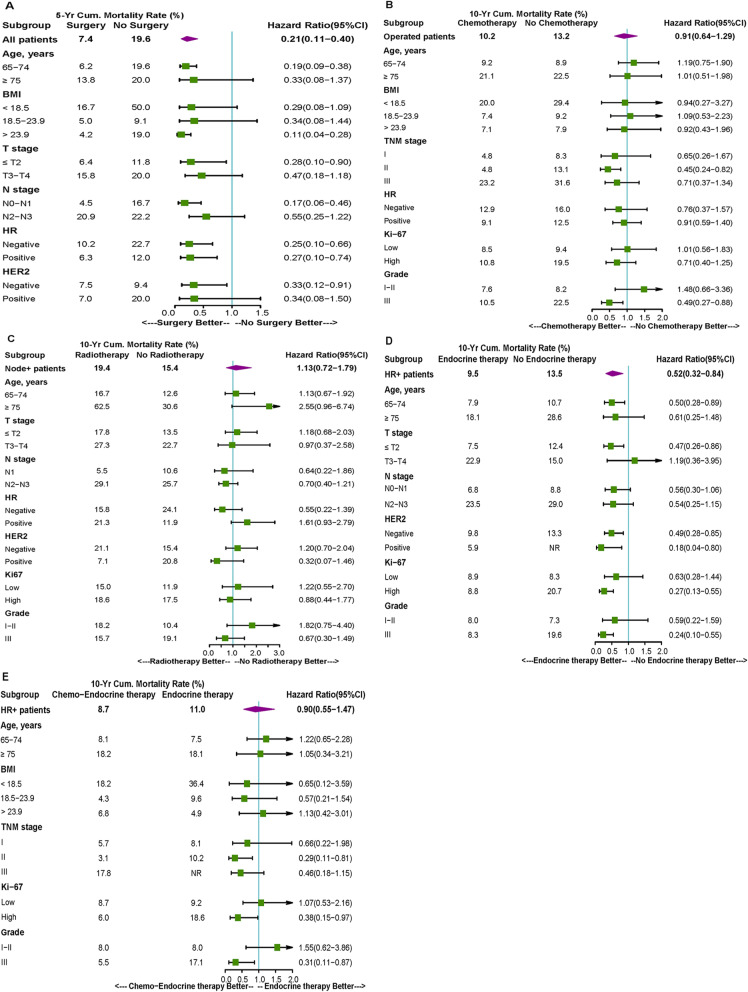
Subgroup analyses of impact of treatment modalities on overall survival. **a**: surgical treatment in all patients; **b**: chemotherapy in post-operative patients; **c**: postmastectomy radiotherapy in patients with lymph node-positive tumors; **d**: endocrine therapy in patients with HR-positive tumors; **e**: chemo-endocrine therapy in patients with HR-positive tumors.

In the survival analysis, patients who underwent surgery had longer survival than those who did not (5-year OS rate: 90.0% vs. 60.0%, *P* <  0.001, Fig. 1a; 5-year DFS rate: 83.8% vs. 61.5%, *P* <  0.001, Additional file 2: Fig. S1A). And there was no significant difference in OS between patients who received modified radical mastectomy and those did radical mastectomy when clinical stage was considered (data not shown). In subgroup analyses, the effect of surgery on OS and DFS in older breast cancer patients was consistently favorable across all subgroups (all HR < 1.0, Fig. 2a and Additional file 2: Fig. S2A). After adjusting for age and clinical stage, surgery was an independent prognostic factor associated with a significant reduction in mortality (HR 0.476, 95% CI 0.250–0.904, *P* = 0.023, Table 4). In addition, surgery was also significantly associated with improved DFS (Additional file 1: Table S2).

**Table 4:** Multivariate analysis of the effect of treatment on overall survival in different population

Treatment	Population	HR^a^ (95% CI)	P-value
Surgery (yes vs. no)	all patients	0.476 (0.250–0.904)	0.023
Adjuvant chemotherapy (yes vs. no)	post-operative patients	0.521 (0.284–0.955)	0.035
Neoadjuvant chemotherapy (yes vs. no)	stage II or III patients	1.430 (0.791–2.587)	0.237
Radiotherapy (yes vs. no)	lymph node+ patients	0.733 (0.415–1.295)	0.285
Endocrine therapy (yes vs. no)	HR+ patients	0.440 (0.261–0.741)	0.002
Targeted therapy (yes vs. no)	HER2+ patients	0.168 (0.029–0.958)	0.045

*HR* hormone receptor, *HER2* human epidermal growth factor receptor 2; HR^a^, hazard ratio; 95% CI, 95% confidence interval

Multivariate analyses were adjusted for age, clinical stage, HR, HER2, tumor grade, treatment types

#### Adjuvant and neoadjuvant chemotherapy

We analyzed the efficacy of adjuvant chemotherapy in older patients who had undergone surgery. Among them, most patients (68.7%) received adjuvant chemotherapy, but elderly group patients were less likely to receive it when compared with those in the older group (35.8% vs 74.7%, *P* <  0.001, Table 3). With regard to the type of chemotherapy, most patients were treated with anthracyclines and/or taxanes, especially for the older group. In Kaplan-Meier analysis, adjuvant chemotherapy seemed to have no effect on OS and DFS (Fig. 1b and Additional file 2: Fig. S1B). However, multivariate analysis showed that adjuvant systemic chemotherapy might be associated with improved survival (OS: HR 0.521, 95% CI 0.284–0.955, *P* = 0.035, Table 4; DFS: Additional file 1: Table S2). In the subgroup analysis, adjuvant chemotherapy could offer significant survival benefit for patients with grade III or stage II disease (OS: HR 0.487, 95% CI 0.269–0.881, *P* = 0.015, Fig. 2b; DFS: Additional file 2: Fig. S2B).

According to current guideline for neoadjuvant chemotherapy in breast cancer, we only assessed the effect of neoadjuvant chemotherapy on older patients staged at II or III (*n* = 734). Among these patients, only 90 (12.3%) cases received neoadjuvant chemotherapy, including 83 (13.2%) cases in the older group and 7 (6.7%) in the elderly group. Neoadjuvant chemotherapy was not associated with survival in multivariate analysis (OS: HR 1.430, 95% CI 0.791–2.587, *P* = 0.237, Table 4; DFS: Additional file 1: Table S2).

#### Radiotherapy

Among the patients who underwent mastectomy, we evaluated the role of adjuvant radiotherapy in older patients with positive-lymph node diseases (*n* = 453). Of these, only 134 (29.6%) patients received postmastectomy radiotherapy, and the older group was more likely to receive adjuvant radiotherapy compared with the elderly group (31.8% vs. 14.0% respectively, *P* = 0.006, Table 3). In the survival analysis, radiotherapy did not confer a benefit in OS, DFS or LRFS on these patients (Fig. 1c and Additional file 2: Fig. S1C). Moreover, receipt of radiotherapy had no impact on OS, DFS and LRFS in all subgroups (Fig. 2c and Additional file 2: Fig. S2C). After adjusting for the potential confounding factors, however, adjuvant radiotherapy following mastectomy showed a significant effect on LRFS, but not OS and DFS. (LRFS: HR 0.247, 95% CI 0.072–0.839, *P* = 0.025; OS: HR 0.733, 95% CI 0.415–1.295, *P* = 0.285, Table 4; DFS: Additional file 1: Table S2).

#### Endocrine therapy

We examined the effect of adjuvant endocrine therapy on survival in older patients with HR-positive breast cancer. Elderly group patients were as likely to be treated with endocrine therapy as those in older group, and the proportion of receiving endocrine therapy were high for both groups (81.7% vs 81.8% respectively, *P* = 0.978, Table 3). Notably, endocrine therapy conferred a significant survival benefit on HR+ patients (Fig. 1d and Additional file 2: Fig. S1D). However, in subgroup analysis, endocrine therapy could provide a significant survival benefit for the older group, but not for the elderly group (Fig. 2d and Additional file 2: Fig. S2D). After adjusting for confounding factors, adjuvant endocrine therapy was indeed an effective treatment option in HR-positive older breast cancer patients (OS: HR 0.440, 95% CI 0.261–0.741, *P* = 0.002, Table 4; DFS: Additional file 1: Table S2).

Stratified analyses were conducted to determine which group of patients would benefit from additional chemotherapy based on endocrine therapy. Among HR-positive patients who received endocrine therapy, patients diagnosed at grade III or with high Ki-67 level were more likely to benefit from additional chemotherapy in terms of OS (OS: HR 0.305, 95% CI 0.107–0.871, *P* = 0.019, Fig. 2e; DFS: Additional file 2: Fig. S2E).

#### Targeted therapy

We analyzed the efficacy of trastuzumab therapy in HER2-positive breast cancer patients (*n* = 143), approximately one-third of patients received trastuzumab treatment. And no difference was observed in the proportion of patients receiving trastuzumab between two groups (Table 3). In the survival analysis, there was no significant difference in survival between patients treated with trastuzumab and those who did not (Fig. 1e and Additional file 2: Fig. S1E). In multivariate analysis, however, receipt of trastuzumab was associated with a lower risk of mortality for HER2-positive patients after adjustment for multiple confounders (OS: HR 0.168, 95% CI 0.029–0.958, *P* = 0.045, Table 4; DFS: Additional file 1: Table S2).

## Discussion

Despite an increase in the number of older breast cancer patients [5], the optimal treatment modality for this population is still a challenging subject. Women aged 65 years and older have been underrepresented in cancer clinical trials [17, 18]. As a consequence, the treatment guidelines for older breast cancer patients are less definitive compared with their younger counterparts due to the paucity of convincing data. Some scholars believe that less intensive treatments for older patients may not decrease their survival compared with standard treatments [7, 19]. However, many published studies showed that less aggressive treatment for older women with early-stage breast cancer seemed to be associated with decreased survival [8, 20]. Where prospective randomized trials are difficult to recruit older patients, a rigorous observational study with a large cohort might be of value for assessing the effect of different therapies on the elderly population, and could provide important guidance for clinical treatment decisions [19]. In the present study, we reviewed the tumor characteristics, real-world treatment patterns and survival outcomes of older patients with early breast cancer at a large tertiary academic medical center.

We comprehensively described the demographic and tumor characteristics of older patients with breast cancer. In this study, older patients more often presented with positive-HR status and negative-HER2 status. This finding was in accord with previous studies, older patients were usually diagnosed with more favorable tumor biology in contrast to young patients [8, 21, 22]. Tumor characteristics were similar between two age groups. However, elderly group patients were more likely to be underweight compared with older group, which implied that functional status declined with age. Moreover, we found that older age at diagnosis was associated with higher risk of mortality, independent of tumor characteristics and treatment. Previous studies also revealed that increasing age was associated with a higher disease-specific mortality [23]. Paradoxically, older women generally have more favorable tumor biology, but they have worse breast cancer–specific mortality. The reasons for this include undertreatment, limited data from clinical trials and reduced age-related immune surveillance [24].

In this cohort, the majority of older patients underwent surgery, even in patients over 75 years of age. And almost all patients received mastectomy rather than breast conserving surgery. Some studies reported that older patients were more likely to receive mastectomy compared with lumpectomy [25, 26], which was consistent with our result. This may be because older patients were less critical of cosmetic appearance, or to avoid radiation therapy. In addition, we found that surgical treatment was associated with better survival, possibly because of a more advanced stage at diagnosis in non-surgery patients. Of the patients who did not receive surgery, 78.4% received chemotherapy. Moreover, survival benefit of surgical treatment in patients older than 80 years have also been confirmed in another study [27], and surgical treatment was safe in older patients.

Adjuvant systemic chemotherapy is an effective treatment modality for young breast cancer patients [28], most patients in our cohort also received adjuvant chemotherapy. However, the proportion of patients treated with chemotherapy decreased with age, which could be explained by the fact that older patients had more comorbidities and concerned about the toxicity of chemotherapy [29, 30]. Limited researches focused on elderly breast cancer patients showed that adjuvant chemotherapy was associated with improved survival in patients at high risk of recurrence [31, 32]. In agreement, our results revealed that adjuvant chemotherapy could confer a survival benefit on patients with grade III or stage II disease. The lack of survival benefit of chemotherapy in stage-III patients may be due to the insufficient number of stage-III patients to produce significant result. Furthermore, we evaluated the role of neoadjuvant chemotherapy in elderly breast cancer patients staged at II-III. The neoadjuvant chemotherapy was less often used and did not offer any benefit to older patients in our cohort, which was seen in previous studies, where neoadjuvant chemotherapy seemed to be an uncommon option and had a lower pathologic complete response rate [33–35].

Postmastectomy radiotherapy (PMRT) was shown to reduce the risks of both recurrence and breast cancer mortality in all patients with node-positive breast cancer considered together [36]. In addition, women aged 70+ years seemed to have similar or higher postmastectomy LRR risks compared to younger women [37]. Therefore, we evaluated the use of PMRT and its impact on older patients with positive-lymph node disease in our cohort. PMRT was given to 31.8% of the patients aged 65 to 74 years, and 14.0% of the patients aged ≥75 years. In clinical practice, there is a prevalent trend and demand to reduce use of postmastectomy radiotherapy in older patients [38]. The decline in use of PMRT among elderly group patients may be due to more comorbidities and concerns about the toxicity of radiotherapy [39]. However, it’s worth noting that PMRT showed a significant effect on LRFS, but not OS and DFS, in older patients with lymph node-positive breast cancer in this study. For women with T1–2 breast cancer and one to two positive axillary lymph nodes, it was also reported that PMRT appeared to be associated with improvement in OS in older patients, but not younger patients [40]. These findings may inform future research direction whether elderly female patients with early breast cancer should receive postmastectomy radiotherapy.

Among HR-positive older patients, the proportion of the elderly group who accepted endocrine therapy was similar to that of older group in our current study. In contrast, several studies reported that women aged > 75 years were less likely to receive endocrine therapy compared with younger patients [41, 42]. As we expected, endocrine therapy might be effective in improving overall survival in older patients with HR+ breast cancer. However, subgroup analysis revealed endocrine therapy could improve OS and DFS in patients aged 65 to 74 years, while patients older than 75 years could not benefit from endocrine therapy. This finding might be caused by lower adherence and shorter duration of endocrine treatment in patients over 75 years old [43]. Furthermore, we found that among HR-positive patients who received endocrine therapy, those with grade III tumors or high Ki-67 level could benefit from additional chemotherapy. It seemed that HR-positive elderly breast cancer patients with high risk of recurrence could benefit from endocrine therapy combined with chemotherapy. Given this result, oncologist may need to consider more treatment choices for elderly HR-positive breast cancer patients with high recurrence risk.

In addition, we described the use and outcome of trastuzumab treatment among older women with HER2-positive breast cancer. Only one third of HER2+ patients received trastuzumab treatment in this cohort, this finding was in line with recent data, they observed that the low utilization of adjuvant trastuzumab for older patients with HER2-positive disease [44]. However, trastuzumab was an effective therapy for HER2-positive breast cancer, with dramatically improved survival, regardless of age [45]. Our study also suggested that use of trastuzumab could reduce the risk of mortality in older patients.

This study had some limitations. Our study did not contain information on treatment-related complications, functional status, patient preference and quality of life, which may affect choice of treatment and survival outcomes. Our findings were based on a large cohort from a single center, thus need further verification in independent data sets.

## Conclusions

In this large cohort, we found that surgical treatment remains a routine and curative option for older patients with early breast cancer. In the adjuvant setting, use of chemotherapy in older patients at high risk of recurrence (grade III or higher stage) was associated with improved survival. And endocrine therapy in HR-positive breast cancer patients and trastuzumab in HER2-positive breast cancer patients may be associated with reduced risk of mortality. We didn’t find neoadjuvant chemotherapy or postmastectomy radiotherapy conferred significant OS or DFS benefit on older breast cancer patients. Moreover, prognostic clinicopathological characteristics should be comprehensively considered when making treatment decisions for those patients.

## Supplementary Information

**Additional file 1: Table S1.** Univariate and multivariate analysis of disease free survival in all breast cancer patients. **Table S2.** Multivariate analysis of the effect of treatment on disease free survival in different population.

**Additional file 2: Fig. S1.** Kaplan–Meier estimates of disease free survival by treatment modalities. **Fig. S2.** Subgroup analyses of impact of treatment modalities on disease free survival.

## Data Availability

The datasets used and/or analysed during the current study are available from the corresponding author on reasonable request.

## Abbreviations

BCIMS: Breast Cancer Information Management System
BMI: Body mass index
CI: Confidence interval
DCIS: Ductal carcinoma in situ
ER: Estrogen receptor
HER2: Human epidermal growth factor receptor-2
HR: Hormone receptor or hazard ratio
IDC: Invasive ductal carcinoma
LRFS: Locoregional recurrence-free survival
LRR: Locoregional recurrence
N stage: Nodal stage
OS: Overall survival
PMRT: Postmastectomy radiotherapy
PR: Progesterone receptor
T stage: Tumor stage
