# Supplementary material for: Treatment patterns and outcomes in older women with early breast cancer: a population-based cohort study in China
Source: BMC Cancer. 2021 Mar 5;21:226. doi: 10.1186/s12885-021-07947-w (PMC7934540; doi:10.1186/s12885-021-07947-w)
Supplement: Supplementary file 1 — Additional file 1: Table S1. Univariate and multivariate analysis of disease free survival in all breast cancer patients. Table S2. Multivariate analysis of the effect of treatment on disease free survival in different population. [file 12885_2021_7947_MOESM1_ESM.docx]

**Treatment Patterns and Outcomes in Older Women with** **Early Breast Cancer:** **A population-based cohort study in China**

**Xu Liu^1^, MD,** **PhD, Dan Zheng^1^, MD, PhD, Yanqi Wu^1^, MD, Chuanxu Luo^1^, MD, PhD, Yu Fan^1^, MD, PhD, Xiaorong Zhong^1,2,^*, MD, PhD, and Hong Zheng^1,2,^*, MD, PhD**

1.Laboratory of Molecular Diagnosis of Cancer, Clinical Research Center for Breast, West China Hospital, Sichuan University, Chengdu, China

2.Department of Head, Neck and Mammary Gland Oncology, Cancer Center, West China Hospital, Sichuan University, Chengdu, China

***Corresponding author:**

Hong Zheng, MD, PhD: hzheng@scu.edu.cn

Xiaorong Zhong, MD, PhD: zhongxiaorong@126.com

Laboratory of Molecular Diagnosis of Cancer, Clinical Research Center for Breast, Department of Head, Neck and Mammary Gland Oncology, Cancer Center, West China Hospital, Sichuan University, 37 Guoxue Xiang, Wuhou District, Chengdu 610041, China, (86)28-8542 2685

| **Table S1** Univariate and multivariate analysis of disease free survival in all breast cancer patients | | | | | |
| --- | --- | --- | --- | --- | --- |
| Variable | Univariate analysis | |  | Multivariate analysis | |
|  | HR^a^ (95% CI) | *P*-value |  | HR^a^ (95% CI) | *P*-value |
| Age, years |  |  |  |  |  |
| 65-74 | 1 |  |  | 1 |  |
| ≥ 75 | 1.620 (1.157 -2.267) | 0.005 |  | 1.556 (0.962-2.518) | 0.072 |
| BMI, kg/m^2^ |  |  |  |  |  |
| 18.5-23.9 | 1 |  |  | 1 |  |
| < 18.5 | 2.765 (1.577-4.848) | < 0.001 |  | 2.538 (1.264-5.097) | 0.009 |
| > 23.9 | 1.322 (0.911-1.920) | 0.142 |  | 1.161 (0.729-1.850) | 0.530 |
| Histology |  |  |  |  |  |
| DCIS | 1 |  |  |  |  |
| IDC | 1.700 (0.921-3.138) | 0.089 |  |  |  |
| Other | 1.243 (0.557-2.776) | 0.595 |  |  |  |
| Clinical stage |  |  |  |  |  |
| 0-I | 1 |  |  | 1 |  |
| II | 1.139 (0.740-1.753) | 0.554 |  | 0.870 (0.460-1.647) | 0.669 |
| III | 4.185 (2.766-6.333) | < 0.001 |  | 3.504 (1.898-6.469) | < 0.001 |
| HR status |  |  |  |  |  |
| Negative | 1 |  |  | 1 |  |
| Positive | 0.704 (0.515-0.963) | 0.028 |  | 0.752 (0.480-1.177) | 0.212 |
| HER2 status |  |  |  |  |  |
| Negative | 1 |  |  |  |  |
| Positive | 1.400 (0.921-2.128) | 0.116 |  |  |  |
| Ki-67 level |  |  |  |  |  |
| Low (≤ 20%) | 1 |  |  | 1 |  |
| High (> 20%) | 1.599 (1.165-2.195) | 0.004 |  | 1.403 (0.886-2.220) | 0.148 |
| Grade |  |  |  |  |  |
| I-II | 1 |  |  | 1 |  |
| III | 1.529 (1.044-2.239) | 0.029 |  | 1.101 (0.691-1.752) | 0.686 |
| Variables with P ≤ 0.05 in univariate analysis were included in the multivariate analysis.  BMI, body mass index; DCIS, ductal carcinoma in situ; IDC, invasive ductal carcinoma; HR, hormone receptor; HER2, human epidermal growth factor receptor 2; HR^a^, hazard ratio; 95% CI, 95% confidence interval. | | | | | |

**Table S2** Multivariate analysis of the effect of treatment on disease free survival in different population

| Treatment | Population | HR^a^ (95% CI) | P-value |
| --- | --- | --- | --- |
| Surgery (yes vs. no) | all patients | 0.474 (0.260-0.863) | 0.015 |
| Adjuvant chemotherapy  (yes vs. no) | post-operative patients | 0.555 (0.355-0.870) | 0.010 |
| Neoadjuvant chemotherapy  (yes vs. no) | stage II or III patients | 1.585 (0.977-2.570) | 0.062 |
| Postmastectomy radiotherapy (yes vs. no) | lymph node+ patients | 0.765 (0.484-1.210) | 0.252 |
| Endocrine therapy  (yes vs. no) | HR+ patients | 0.527 (0.336-0.828) | 0.005 |
| Targeted therapy  (yes vs. no) | HER2+ patients | 0.262 (0.071-0.961) | 0.043 |
| HR, hormone receptor; HER2, human epidermal growth factor receptor 2; HR^a^, hazard ratio; 95% CI, 95% confidence interval.  Multivariate analyses were adjusted for age, clinical stage, HR, HER2, Ki-67, tumor grade, treatment types. | | | |
