# Supplementary material for: Treatment patterns and outcomes in older women with early breast cancer: a population-based cohort study in China
Source: BMC Cancer. 2021 Mar 5;21:226. doi: 10.1186/s12885-021-07947-w (PMC7934540; doi:10.1186/s12885-021-07947-w)
Supplement: Supplementary file 2 — Additional file 2: Fig. S1. Kaplan–Meier estimates of disease free survival by treatment modalities. Fig. S2. Subgroup analyses of impact of treatment modalities on disease free survival. [file 12885_2021_7947_MOESM2_ESM.pdf]

Treatment Patterns and Outcomes in Older Women with Early Breast Cancer: A population-based cohort study in China

Xu Liu[1], MD, PhD, Dan Zheng[1], MD, PhD, Yanqi Wu[1], MD, Chuanxu Luo[1], MD, PhD, Yu Fan[1], MD, PhD, Xiaorong Zhong[1,2,*], MD, PhD, and Hong Zheng[1,2,*], MD, PhD

1.Laboratory of Molecular Diagnosis of Cancer, Clinical Research Center for Breast, West China Hospital, Sichuan University, Chengdu, China

2.Department of Head, Neck and Mammary Gland Oncology, Cancer Center, West China Hospital, Sichuan University, Chengdu, China

***Corresponding author:**

Hong Zheng, MD, PhD: hzheng@scu.edu.cn

Xiaorong Zhong, MD, PhD: zhongxiaorong@126.com

Laboratory of Molecular Diagnosis of Cancer, Clinical Research Center for Breast, Department of Head, Neck and Mammary Gland Oncology, Cancer Center, West China Hospital, Sichuan University, 37 Guoxue Xiang, Wuhou District, Chengdu 610041, China, (86)28-8542 2685

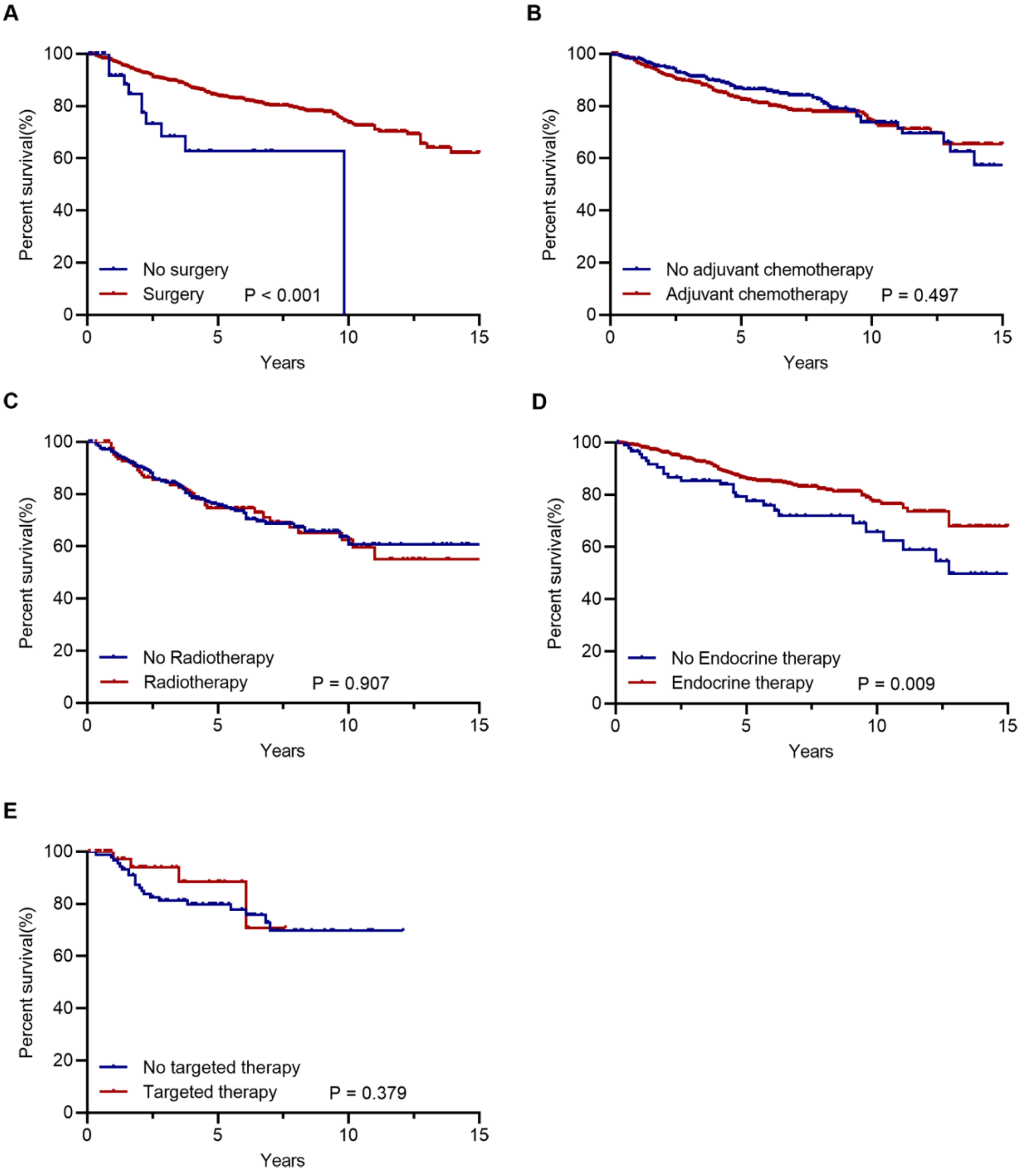

Fig S1 Kaplan–Meier estimates of disease free survival by treatment modalities

A: surgical treatment in all patients; B: chemotherapy in post-operative patients; C: postmastectomy radiotherapy in patients with lymph node-positive tumors; D: endocrine therapy in patients with HR-positive tumors; E: targeted therapy in patients with HER2-positive tumors.

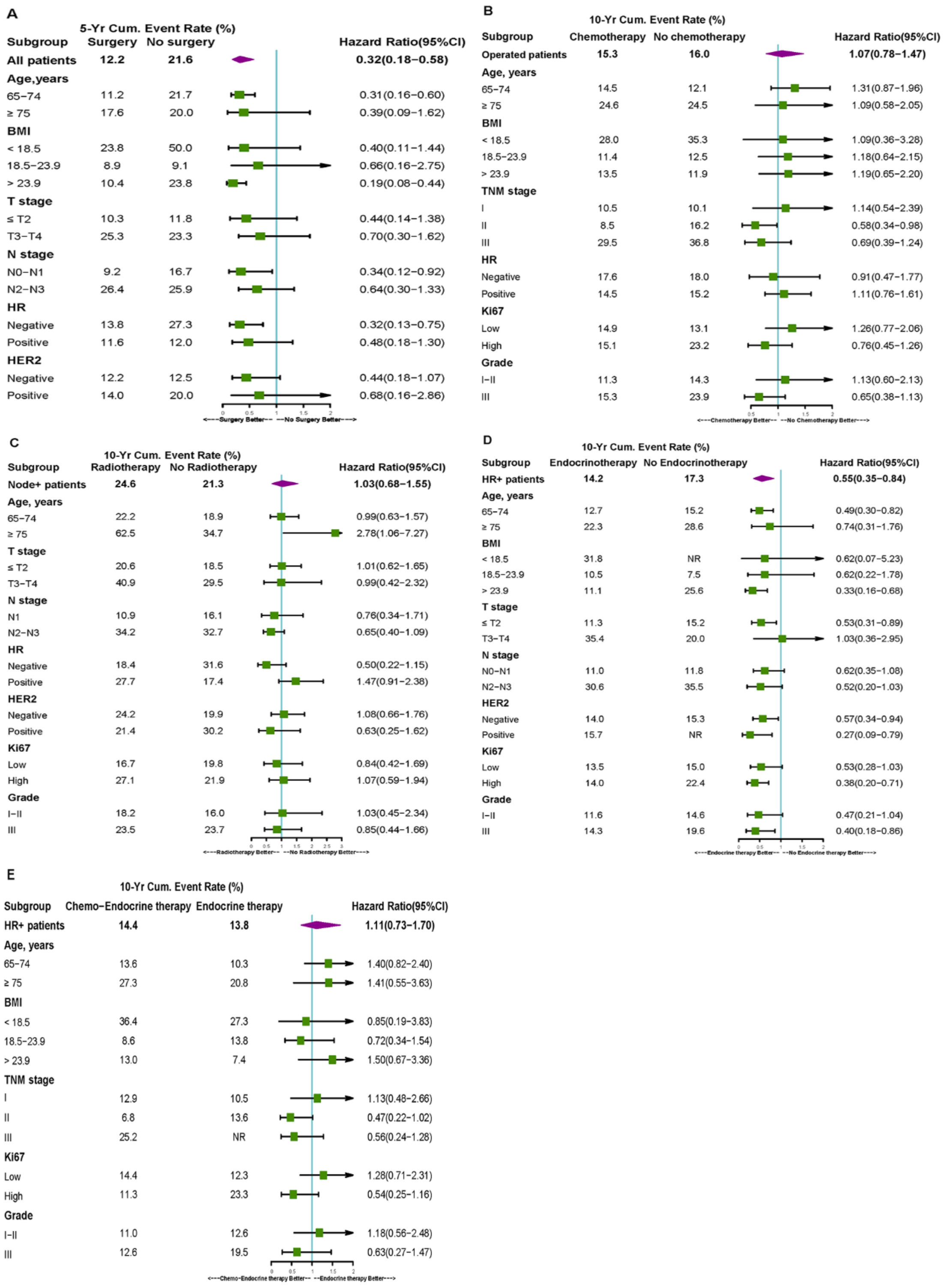

Fig S2 Subgroup analyses of impact of treatment modalities on disease free survival

A: surgical treatment in all patients; B: chemotherapy in post-operative patients; C: postmastectomy radiotherapy in patients with lymph node-positive tumors; D: endocrine therapy in patients with HR-positive tumors; E: chemo-endocrine therapy in patients with HR-positive tumors.
